# Bacterial Growth Kinetics under a Novel Flexible Methacrylate Dressing Serving as a Drug Delivery Vehicle for Antiseptics

**DOI:** 10.3390/ijms140510582

**Published:** 2013-05-21

**Authors:** Christina Forstner, Johannes Leitgeb, Rupert Schuster, Verena Dosch, Axel Kramer, Keith F. Cutting, David J. Leaper, Ojan Assadian

**Affiliations:** 1 Department of Medicine I, Division of Infectious Diseases and Tropical Medicine, Medical University of Vienna, Waehringer Guertel 18-20, 1090 Vienna, Austria; E-Mail: christina.a.forstner@meduniwien.ac.at; 2 Department for Trauma Surgery, Medical University of Vienna, Waehringer Guertel 18-20, 1090 Vienna, Austria; E-Mails: johannes.leitgeb@meduniwien.ac.at (J.L.); rupert.schuster@meduniwien.ac.at (R.S.); 3 Department for Hospital Hygiene, Medical University of Vienna, Waehringer Guertel 18-20, 1090 Vienna, Austria; E-Mail: verena.dosch@meduniwien.ac.at; 4 Institute for Hygiene and Environmental Medicine, University Medicine Greifswald, Walther-Rathenau Strasse 49a, 17489 Greifswald, Germany; E-Mail: kramer@uni-greifswald.de; 5 Faculty of Society and Health, Buckinghamshire New University, WD3 5TG Uxbridge, UK; E-Mail: kc@healthdirections.co.uk; 6 Faculty of Medical Sciences, Newcastle University, NE1 7RU Newcastle upon Tyne, UK; E-Mail: profdavidleaper@doctors.org.uk

**Keywords:** wound dressing, biofilm, drug delivery, antiseptic, polyhexanide, povidone-iodine, octenidine-dihydrochloride, methacrylate, Altrazeal, Prontosan C, Octenisept, Braunol

## Abstract

A flexible methacrylate powder dressing (Altrazeal^®^) transforms into a wound contour conforming matrix once in contact with wound exudate. We hypothesised that it may also serve as a drug delivery vehicle for antiseptics. The antimicrobial efficacy and influence on bacterial growth kinetics in combination with three antiseptics was investigated in an *in vitro* porcine wound model. Standardized *in vitro* wounds were contaminated with *Staphylococcus aureus* (MRSA; ATCC 33591) and divided into six groups: no dressing (negative control), methacrylate dressing alone, and combinations with application of 0.02% Polyhexamethylene Biguanide (PHMB), 0.4% PHMB, 0.1% PHMB + 0.1% betaine, 7.7 mg/mL Povidone-iodine (PVP-iodine), and 0.1% Octenidine-dihydrochloride (OCT) + 2% phenoxyethanol. Bacterial load per gram tissue was measured over five days. The highest reduction was observed with PVP-iodine at 24 h to log_10_ 1.43 cfu/g, followed by OCT at 48 h to log_10_ 2.41 cfu/g. Whilst 0.02% PHMB resulted in a stable bacterial load over 120 h to log_10_ 4.00 cfu/g over 120 h, 0.1% PHMB + 0.1% betaine inhibited growth during the first 48 h, with slightly increasing bacterial numbers up to log_10_ 5.38 cfu/g at 120 h. These results indicate that this flexible methacrylate dressing can be loaded with various antiseptics serving as drug delivery system. Depending on the selected combination, an individually shaped and controlled antibacterial effect may be achieved using the same type of wound dressing.

## 1. Introduction

An infection occurs, following contamination and colonization by bacteria, when a wound and surrounding tissue is invaded with a subsequent inflammatory reaction. An infection may remain localized or may generalize. The occurrence of wound infection depends on the underlying condition of the patient and associated immune response, the amount of primary contamination and on the virulence of the involved bacteria [1].

Wound infection may impede the process of healing and eventually lead to severe and life threatening complications. Despite low prevalence of multi-resistant Gram-positive pathogens in Austria, wounds have been identified as primary source of infection in at least 1.6% of hospitalized patients with multi-resistant *Staphylococcus aureus* (MRSA) bacteremia [2]. A study conducted in Brazil demonstrated that in patients with pressure ulcers stage II 65% of wounds were colonized or infected with multidrug-resistant organisms, thereof 33% with Gr-negative bacilli, 21% with MRSA, and 47% with both [3]. Therefore, promoting the healing process and protecting a wound from potential contamination with pathogenic and/or multi-resistant bacteria is imperative for prevention of wound infection. Wound dressing selection depends on the type of wound and its etiology, its severity, its condition, particularly the level of contamination and infection, and its anatomic location. However, all dressings are intended to protect a wound from mechanical and environmental injuries, from microbial contamination, and eventually to promote wound healing. Over the past three decades, an increasing number of different wound dressing types and categories have been developed. In brief, modern wound dressings may be categorized as film, pad dressings with a functional or non-adherent island, adherent, or moist dressings such as hydrogel and hydrocolloid dressings, and absorbent dressings such as alginates, foam dressings, or super-absorbing dressings [4] In addition, wound dressings may be non-antimicrobial or may have an antimicrobial component, such as povidone iodine, chlorhexidine, silver or polyhexamethylene biguanide (PHMB) [5].

Today, and particularly in light of a global increase of antibiotic resistant bacteria [6,7], antimicrobial dressings can play an important role in wound care in the prevention and management of infection. While universal agreement on clinical indicators relevant to intervention with antimicrobial dressings remain illusive [8,9], it is important to remember that all antimicrobial wound dressings have different physical and pharmacological properties. These include: the type and amount of antimicrobial they release, the duration and mode of antimicrobial action, the dressing’s ability to manage varying volumes of wound exudate, to decrease malodour or pain. Apart from the specific, physical characteristics of different antimicrobial dressings, the most widely used agents in antimicrobial dressings are various forms of silver (ionic, elementary, nano-crystalline, or silver salts) [10], iodine [11], and increasingly medical honey. Some antimicrobial dressings contain PHMB [12,13], others chlorhexidine, and so far, no commercially available wound dressing contains the antiseptic octenidine-dihydrochloride (OCT) [14] yet. The available range of these different dressings in clinical practice means that large volumes of dressings need to be kept in stock. Furthermore, medical care providers are restricted in their practice to limited numbers of antimicrobial compounds, and have little possibility to alter the pharmacokinetic release profile of the applied antimicrobial. Therefore, a dressing would be beneficial, which allows medical professionals to decide whether the dressing shall be applied as an antimicrobial or non-antimicrobial dressing, and which antimicrobial compound should be used.

Recently, a flexible methacrylate dressing (Altrazeal^®^, Uluru Inc., Addison, TX, USA) has been introduced [15]. This dressing, which comes as a powder and transforms into a wound contour conforming matrix once in contact to wound exudate, is based on dehydrated hydrogel modified into 60–65 μm small particles containing a poly-2-hydroxyethyl-/poly-2-hydroxypropyl (pHEMA/ pHPMA) -methacrylate backbone and terminal hydroxyl group. This polymer is a member of the family of hydrophilic polymers that contain a covalent methacrylate backbone with a hydroxyl aliphatic side chain, resembling very much the polymers used in soft contact lenses. The dressing can be directly applied into a wound and transforms in presence of wound exudate, or can be hydrated with saline or other sterile solutions, resulting in transformation into a wound contour conforming flexible dressing. After aggregation, capillary channels of approximately 7 nm width wick exudate from the wound surface through a high moisture vapor transpiration rate, of approximately 12,000 mL/m^2^/24 h, whilst at the same time promoting a moist wound environment for healing [16]. The aggregated dressing contains approximately 68% water, which is similar to the water content of the skin (72%–74%), further increasing its biological compatibility.

Because of the inert nature of the aggregated polymer whereby only the pHEMA/pHPMA-methacrylate particles react with each other, we hypothesised that this novel dressing may be an ideal candidate to serve as a drug delivery vehicle for various cationic and anionic wound antiseptics. Therefore, the antimicrobial efficacy of this dressing, in combination with five antimicrobial wound irrigation solutions/antiseptics, and its combined influence on bacterial growth kinetics was investigated in this study using an *in vitro* wound model.

## 2. Results and Discussion

### 2.1. Results for Negative Control and Non-Antimicrobial Methacrylate Dressing

Without any dressing, the mean *Staphylococcus aureus* load per gram tissue started at log_10_ 4.02 cfu/g tissue, and followed a typical bacterial growth curve increasing to a mean load of log_10_ 7.07 cfu/g tissue at 120 h. Without loading of any antimicrobial compound the methacrylate dressing did not prevent bacterial growth; however, it decreased multiplication of bacterial numbers per gram tissue to the magnitude of 1.5 log_10_ compared with the negative control.

### 2.2. Results for Methacrylate Dressing Loaded with Antimicrobial Compounds

The highest bacterial reduction was observed with PVP-iodine at 24 h to log_10_ 1.43 cfu/g tissue, followed by OCT at 48 h to log_10_ 2.41 cfu/g tissue.

In combination with PHMB the dressing resulted in different bacterial kinetics: 0.02% PHMB resulted in a stable bacterial load at a mean log_10_ 4.00 cfu/g tissue over 120 h; 0.1% PHMB + 0.1% betaine inhibited growth during the first 48 h, with slightly increasing bacterial numbers up to log_10_ 5.38 cfu/g tissue at 120 h; 0.4% PHMB resulted in a kinetic curve similar to OCT with approximately 0.5–1.5 log_10_ higher bacterial counts over all time periods. Detailed results are summarized in Table 1.

**Figure 1:**
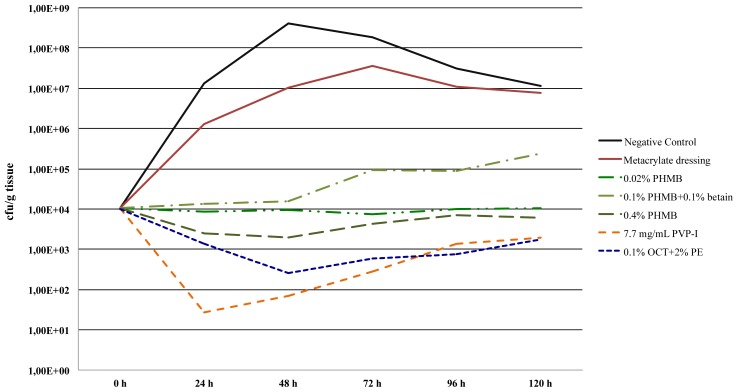
Kill-time study and bacterial growth kinetics of *S. aureus* (ATCC 33591) in presence and absence of a methacrylate dressing with and without single loading of antiseptics over five days application. Bacterial counts expressed as absolute numbers per gram of tissue. Data represent the means of four independent experiments each after standard contamination with 1.0 × 10^7^ cfu *S. aureus* per gram tissue. Results are depicted using absolute numbers.

The data indicate that the test conditions were stable and reproducible. In particular, the initial bio-burden per gram of tissue after contamination with an inoculum of log_10_ 7.00 cfu/g yielded a comparable *S. aureus* load of approximately log_10_ 4.00 cfu/g tissue in all test essays. Although the individual results seem to show a wide distribution, and particularly for the negative control assay with higher individual cfu-counts, the standard deviations always ranged 1–2 log_10_ below the respective mean results. Such variations are frequently observed in biological systems [17–20] and are to be expected. Most importantly, the results of this experimental study establish the possibility to load a methacrylate dressing with various antiseptics, which are released into the surrounding tissue. The presence of the antimicrobial compound in tissue was indirectly demonstrated by the reduced bacterial counts per gram of tissue, as no other possibility exists explaining the lower cfu/ g tissue counts of *S. aureus* in the respective essays. The detailed bacterial growth kinetics are shown in Figure 1.

Interestingly, the use of methacrylate polymers in wound care is not totally new and was proposed some three decades earlier. Migliaresi *et al.* [21] reported on a polybutadiene layer to which poly(2-hydroxyethyl methacrylate) (pHEMA) was thermally grafted. The authors suggested the use of such compound materials to be used as burn wound cover, since the presence of polybutadiene increased the tensile properties of the dressing and prevented water diffusion while it did not affect the oxygen permeability of the pHEMA. The use of pHEMA with other admixtures such as polyethylene glycol-400 (PEG), including antimicrobial additives such as silver sulfadiazine, silver nitrate, gentamicin or nitrofurazone in burn wounds was also proposed [22]. Experimental and clinical studies showed that adding antimicrobial compounds to a pHEMA base did not only reduce the bacterial count in burn wounds, but also exhibited a pain release effect in patients [22–24]. After some years of absence, methacrylate polymers shift again into attention as material base for wound dressings. Recently, electrospinned zwitterionic poly(sulfobetaine methacrylate) (pSBMA) was investigated to be used as a superabsorbent and non-adherent wound dressing [25], including its used with antimicrobial compounds [26,27]. However, while promising, all these previous approaches have either used woven polymers in form of a 2-dimensional dressing or in form of a composite gel. The investigated methacrylate dressing studied in our experiments goes one step further since it can be applied directly into the wound bed as a pure and inert pHEMA/ pHPMA powder, which than transforms into a polymer matrix allowing complete wound bed contouring and easy removal.

However, there are some aspects which need to be highlighted. Firstly, the kill-time curves represent the bacterial growth kinetics in non-vital and undisturbed tissue. Influencing conditions in clinical wounds may be different, particularly pertaining to mechanical pressure, temperature, and surrounding pH. Secondly, it would be a misinterpretation to conclude that PVP-iodine is the most effective antiseptic, or that 0.1% PHMB with 0.1% betaine is the least effective antimicrobial compound in this study. Selection of antiseptics in wound care depend on a number of factors such as: identification of pathogens, the clinical condition of the patient and the etiology of a specific wound, and the health care provider’s intention to apply an antiseptic therapeutically, either to treat a localized infected wound, or to prevent a wound from becoming infected. In this regard, the methacrylate dressing may be useful in not only serving as a wound dressing, but also as a drug delivery matrix for the most appropriate antimicrobial compound. Hence, the dressing permits application as a non-antimicrobial dressing, if antimicrobial action is not required. It also may be used as an antimicrobial dressing [28] to protect a non-infected wound from infection by loading it with, for example 0.1% PHMB + 0.1% betaine, or to eradicate colonizing microorganisms with, for example 0.4% PHMB, or to treat infected wounds by rapid kill of pathogenic microorganisms with, for example OCT or PVP-iodine. Following this algorithm, a health care provider may offer patients an individual, tailored, wound treatment based on individual clinical demands.

Thirdly, it may be assumed that many of the bacteria in the model may have been attached to tissue and eventually may have formed biofilms, which reduces their antimicrobial susceptibility. Therefore, the antimicrobial efficacy of various antiseptics in tissue was lower than what is seen in quantitative suspension tests, where antiseptics get in contact to planktonic bacteria instead of their sessile form. Yet, the results of the direct bacterial reduction indicate that a methacrylate dressing loaded with antiseptics may have an effect even on sessile bacteria embedded in biofilm. However, the limitation of the experiment was that presence of biofilms was not demonstrated by additional staining techniques.

And finally, the experimental design of this study investigated only the kill-time kinetic after one single application of an antiseptic. Repeated administration of antiseptics most likely would have yielded for all tested compounds far lower bacterial counts over all time points. However, the clinical relevance of such strategy for wound care management to use a methacrylate dressing loaded with antiseptics remains unknown and requires future research.

## 3. Experimental Section

### 3.1. *In-Vitro* Test Model

For the *in vitro* test model, fresh raw porcine meat of comparable size (30 × 30 × 30 cm) was used for each experiment. A standard wound of 4 × 4 × 1 cm was cut into the pork meat using sterile instruments and maintaining aseptic conditions. The wound was then experimentally contaminated with a bacterial test-suspension and incubated for 8 h at 35 °C.

### 3.2. Preparation of the Test-Suspension and Artificial Contamination of the Wound

For the test-suspension, a clinical isolate of a well described Methicillin-resistant *Staphylococcus aureus* (MRSA; strain ATCC 33591) was used. From an overnight culture on Brain Heart Infusion (BHI) agar plates an optical density (OD) of McFarland 0.5 was set using a VITEK 2 Densi-check (bioMérieux, Marcy l'Etoile, France). Using sterile 0.9% NaCl as suspension solution, the final bacterial count was set to 1.0 × 10^7^ (log_10_ 7.00) cfu/mL. The artificial wounds were then contaminated with 1 mL of the test-suspension and incubated at 37 ± 1 °C for 8 h. After the incubation period the rest of the test-suspension was removed without contaminating the surrounding tissue and 4 biopsies of the wounds (each 5 mm diameter, 5 mm depth) were taken, resulting in a reproducible wound colonization of 1.0 × 10^4^ (log_10_ 4.00) cfu/g of tissue (Table 1).

### 3.3. Preparation of Test Model and Processing of Tissue Biopsies

Wounds were treated with no dressing (negative control), the methacrylate test dressing (Altrazeal^®^; Uluru Inc., Addison, TX, USA) alone, and in combination with 0.02% PHMB (20% Cosmocil^®^; Arch Chemicals Inc., Norwalk, CT, USA), 0.1% PHMB + 0.1% betaine (Prontosan C^®^; B. Braun Melsungen AG, Melsungen, Germany), 0.4% PHMB (Lavasept^®^; B. Braun Melsungen AG, Melsungen, Germany), 7.7 mg/mL PVP-iodine (Braunol^®^; B. Braun Melsungen AG, Melsungen, Germany), and 0.1% OCT + 2% phenoxyethanol (Octenisept^®^; Schülke & Mayr GmbH, Norderstedt, Germany), respectively.

Bacterial loads per gram of tissue were measured after 0 h, 24 h, 48 h, 72 h, 96 h and 120 h. All experiments were conducted in quadruplicate. One wound without any dressing served as negative control to assess the normal bacterial kinetic of the *in vitro* wound model. After removal of the test dressing, four tissue biopsies each were taken, weighed and homogenized using 0.9% NaCl in addition to 0.6 μg/mL oxacillin. Bacterial numbers in the homogenized solutions were then determined using microtiter-plates filled with BHI broth containing 75 mg/mL aztreonam. The number of bacteria was noted as absolute count or log_10_ cfu/g tissue, whenever appropriate. The means together with standard deviation (± SD) were calculated and set as the starting point for the wound colonization.

## 4. Conclusions

This study demonstrates that a methacrylate dressing applied into an experimental wound as a powder, which then transforms into a flexible, wound-contouring dressing, can be combined with a number of antiseptics and serve as a drug delivery system for antimicrobial compounds. Depending on the selected moisturizing solution, an individually tailored and controlled non-antimicrobial or antimicrobial effect may be achieved using the same type of wound dressing. This opens new possibilities for topical antimicrobial treatment and prophylactic strategies in wound care management. It needs to be elucidated in future studies, as to whether these effects correlate with the clinical (*in vitro*) situation in infected or contaminated wounds.

## Figures and Tables

**Table 1:** Kinetics of *S. aureus* (ATCC 33591) under a methacrylate dressing alone and in combination with five antiseptics over five days after a single application of the antimicrobial compound.

Dressing +/− antiseptic or no dressing	Mean bacterial colony forming units (log_10_) per gram tissue (±1 SD) after					

	0 h	24 h	48 h	72 h	96 h	120 h
Negative control	4.02 (± 2.67)	7.12 (± 6.52)	8.61 (± 7.43)	8.26 (± 7.12)	7.56 (± 6.59)	7.07 (± 6.89)
Methacrylate dressing	4.02 (± 2.67)	6.11 (± 5.40)	7.02 (± 7.11)	7.56 (± 6.71)	7.05 (± 5.80)	6.90 (± 6.65)
0.02% PHMB a	4.01 (± 2.46)	3.93 (± 2.85)	3.98 (± 2.93)	3.87 (± 2.94)	4.01 (± 2.83)	4.01 (± 2.71)
0.1% PHMB a + 0.1% betaine b	4.03 (± 2.67)	4.12 (± 3.63)	4.20 (± 3.54)	4.96 (± 3.76)	4.95 (± 3.71)	5.38 (± 4.96)
0.4% PHMB a	4.01 (± 2.32)	3.39 (± 2.68)	3.29 (± 2.10)	3.63 (± 2.87)	3.84 (± 2.35)	3.79 (± 2.26)
7.7 mg/mL PVP-I c	4.02 (± 2.67)	1.43 (± 0.91)	1.84 (± 1.01)	2.45 (± 1.64)	3.14 (± 1.98)	3.29 (± 1.89)
0.1% OCT d + 2% PE e	4.01 (± 1.91)	3.14 (± 1.79)	2.41 (± 1.69)	2.78 (± 1.49)	2.89 (± 1.74)	3.24 (± 2.02)

a PHMB = Polyhexamethylene Biguanide;

b Betaine = Undecylenamidopropyl-Betain;

c PVP-I = Povidone-iodine;

d OCT = Octenidine-dihydrochloride;

e PE = Phenoxyethanol; Data represent the means (±1 SD) of four independent experiments each after standard contamination with log_10_ 7.00 cfu S. aureus per gram tissue.
